# How the Gus Schumacher Produce Prescription Program Works: An Adaptation of a Nutrition Incentive Theory of Change

**DOI:** 10.3390/nu15153352

**Published:** 2023-07-28

**Authors:** Sarah A. Stotz, Nadine Budd Nugent, Melissa Akers, Kirsten Leng, Carmen Byker Shanks, Amy L. Yaroch, James Krieger, Morgan Szczepaniak, Hilary Seligman

**Affiliations:** 1 Centers for American Indian and Alaska Native Health, Colorado School of Public Health, University of Colorado Anschutz Medical Campus, Aurora, CO 80045, USA; 2 Gretchen Swanson Center for Nutrition, Omaha, NE 68154, USA; dinabuddnugent@gmail.com (N.B.N.); cbshanks@centerfornutrition.org (C.B.S.); ayaroch@centerfornutrition.org (A.L.Y.); mbahl@centerfornutrition.org (M.S.); 3 Division of General Internal Medicine, University of California, San Francisco, CA 94143, USA; melissa.akers@ucsf.edu (M.A.); hilary.seligman@ucsf.edu (H.S.); 4 Center for Vulnerable Populations, San Francisco General Hospital, San Francisco, CA 94110, USA; 5 Healthy Food America, Seattle, WA 98122, USA; kirsten.leng@yahoo.com (K.L.); jkrieger@hfamerica.org (J.K.); 6 School of Public Health, University of Washington, Seattle, WA 98195, USA

**Keywords:** Gus Schumacher Nutrition Incentive Program (GusNIP), produce prescription, low income, food insecurity, theory of change, nutrition security

## Abstract

The United States Department of Agriculture’s Gus Schumacher Nutrition Incentive Program (GusNIP) supports nutrition incentive (NI) and produce prescription programs (PPRs). PPRs allow healthcare providers to “prescribe” fruits and vegetables (FVs) to patients experiencing low income and/or chronic disease(s) and who screen positive for food insecurity. We developed a Theory of Change (TOC) that summarizes how and why PPRs work, identifies what the programs hope to achieve, and elucidates the causal pathways necessary to achieve their goals. We created the PPR TOC through an iterative, participatory process that adapted our previously developed GusNIP NI TOC. The participatory process involved food and nutrition security experts, healthcare providers, PPR implementors, and PPR evaluators reviewing the existing NI TOC and suggesting modifications to accurately reflect PPRs. The resulting TOC describes the mechanisms, assumptions, rationale, and underpinnings that lead to successful and equitable outcomes. Modifications of the NI TOC centered around equity and focused on inclusion of healthcare as an additional partner and the importance of health and healthcare utilization as outcomes. The TOC describes how the GusNIP PPR program reaches its goals. This understanding will be useful for PPR developers, implementers, funders, and evaluators for describing the pathways, assumptions, and foundations of successful PPRs.

## 1. Introduction

Fruits and vegetables (FVs) are a critical part of a healthy diet. Inadequate FV intake is associated with the development of obesity, type 2 diabetes, hypertension, cardiovascular disease, stroke, and some types of cancers [1,2,3,4,5,6]. These diet-related chronic diseases reduce quality of life and are among the leading causes of death in the United States (U.S.) [7]. Moreover, poor diets and related health complications cost the U.S. healthcare system over USD 50 billion annually [8].

The 2020–2025 Dietary Guidelines for Americans recommend that adults consume the equivalent of five servings of FVs per day, yet only one in ten adults in the U.S. meet this recommendation [9]. Further, FV intake is lowest among U.S. adults with a low income due to long-standing systemic inequities in the food system [10].

Increasing FV intake is difficult for many Americans [11]. Availability and access to FVs are major influences on choice and, ultimately, intake [12,13,14,15]. Access to high-quality FVs is lower in rural and low-income communities and communities of color [16,17]. The price of food is a critical determinant of consumer purchasing decisions [18,19,20,21]. FVs are expensive relative to other food groups [22,23], with pricing higher than many households with low income can afford. While individuals ultimately make the choice to consume certain foods and beverages, the current food environment exploits biological, psychological, and socio-economic vulnerabilities that encourage the intake of energy-dense, nutrient-poor options over healthier ones such as FVs [24].

Financial incentives for FVs are an evidence-based strategy to increase FV purchases and consumption among households who experience low income [25,26,27]. Financial incentives for FVs are hypothesized to positively impact individual dietary intake and health, healthcare costs, and local economic growth [25,26,27]. Intervention studies have demonstrated that price reductions can positively affect consumer demand and consumption of healthful foods like FVs [28,29,30,31]. Financial incentives nudge consumers towards healthier choices by reducing the cost of targeted food [31]. Incentives are often provided as a discount or voucher to apply towards the purchase of FVs, ultimately enhancing an individual’s ability to access, afford, and purchase healthy foods [32,33,34,35,36,37].

Federally supported financial incentive programs were first introduced in the 2008 Farm Bill through the Healthy Incentives Pilot (HIP), which tested the impact of incentives on the FV intake of SNAP households in Hampden County, Massachusetts [38]. This randomized controlled trial found that providing households with a 30% discount on FVs led to an increase in FV intake by over ¼ cup per day compared to the control group [38]. In the 2014 Farm Bill, Congress funded the Food Insecurity Nutrition Incentive program (FINI) at USD 100 million across five years. FINI provided funding to 41 states to implement nutrition incentive (NI) programs [39]. The most recent 2018 Farm Bill replaced FINI with the larger Gus Schumacher Nutrition Incentive Program (GusNIP) at USD 250 million over five years [40]. Subsequently, Congress added USD 69 million through the GusNIP COVID Relief and Response (GusCRR) grants program in 2021 and USD 40 million through the American Rescue Plan Act (ARPA) in 2022 to support more GusNIP programs. The U.S. Department of Agriculture, National Institute of Food and Agriculture (USDA NIFA) administers funding for GusNIP grants [40].

GusNIP supports two types of financial incentive programs: nutrition incentives and produce prescriptions. NI programs focus on increasing the purchase and intake of FVs among participants who use SNAP. Typically, participants who use SNAP receive incentives (e.g., vouchers, coupons, automatic discounts) to purchase FVs when shopping at participating retailers (e.g., farmers markets, grocery stores). For instance, SNAP participants who purchase USD 10 of FVs may receive USD 20 worth of FVs at participating sites [41].

Produce prescription programs (PPRs) are a second type of financial incentive that are available through GusNIP. The population eligible to enroll in PPRs are those who are experiencing low income and/or chronic disease(s), such as type 2 diabetes, and screen positive for food insecurity within a healthcare system. Healthcare providers “prescribe” FVs to patients using vouchers (or a similar mechanism), which can be redeemed for FVs at participating food retailers or clinic-based food “farmacies [41]”.

Studies of PPRs have shown modest increases in FV intake, decreases in food insecurity, and a possible protective effect on the development and course of certain diet-related chronic diseases [41,42,43,44]. While evidence on PPRs is building rapidly, the methodology of existing studies is relatively weak, so caution is warranted in drawing causal conclusions on their effectiveness [33,34,35]. Theoretical frameworks to situate these programs are needed to guide stronger program evaluation, rigorous study design, and funding to support evaluation.

GusNIP also funds the Nutrition Incentive Program Training, Technical Assistance, Evaluation, and Information Center (NTAE) [45,46], which supports GusNIP applicants and grantees in reporting, evaluation, and technical assistance [45,46]. In 2021, the GusNIP NTAE, its partners, grantees, and participants developed a Theory of Change (TOC) for the GusNIP NI program [47]. A TOC describes how and why a program works to bring about a desired change or outcome [48,49,50,51]. The TOC process first identifies what a program hopes to achieve, the intermediate outcomes that are necessary to reach the goal, and finally, the program activities that lead to the outcomes and the causal pathways linking them. A TOC also describes the environment in which a program operates and assumptions about existing conditions that are already in place and necessary for program success. A TOC is developed using a structured and participatory process that includes a review of current evidence and program documents, as well as interactive input from people implementing and benefitting from the program and from program partners, such as participating food retail organizations (e.g., grocery stores, farmers markets, etc.). A TOC communicates to funders, advocates, program implementers, partners, and others how a program works; provides a framework for program development, growth, and evaluation; and creates a shared program understanding among partners. A TOC is a living, evolving theory that is refined as new evidence emerges, the environment changes, and experience is gained [48,49,50,51,52].

To date, no PPR TOC exists. A PPR TOC will help clinical systems and their partners, participants, and funders build upon an existing program framework and implement a model that is based in evidence. To address this gap and need, this article focuses on the development and presentation of a PPR TOC to establish a shared understanding of how PPRs work.

## 2. Methods

**Figure 1:**
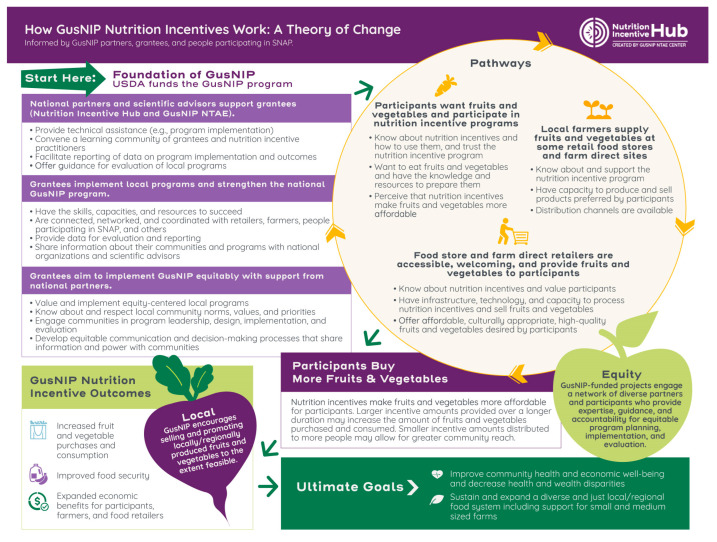
GusNIP Nutrition Incentive Theory of Change [47].

The GusNIP NI TOC served as a foundation upon which the PPR TOC was adapted and built, through a three-step PPR TOC development process. Leng et al. provide a detailed description of the GusNIP NI TOC methodology [47]; a summary follows. Further, the NI TOC graphic can be found in Figure 1.

The GusNIP NI TOC was developed collaboratively with GusNIP NTAE staff members and partners; agriculture, food retail, academic, anti-hunger, and nutrition sectors; other NI practitioners; and community members using a retrospective, equity-centered, participatory process. Details on these methods can be found elsewhere [47]. Process facilitators, informed by GusNIP documents and a literature review, developed an initial PPR TOC draft. Next, a facilitator interviewed partners and practitioners to introduce them to the TOC concept and identify key TOC elements and then used thematic analysis to develop the next TOC draft. Partners and NI practitioners refined this draft over the course of three workshops. Refinement included using the most salient themes from thematic analysis of each workshop in an iterative manner [47]. Facilitators conducted three focus groups with community members who had used nutrition incentives to ground the draft TOC in participants’ lived experiences. Finally, the facilitators integrated all this information into a final NI TOC.

**Figure 2:**
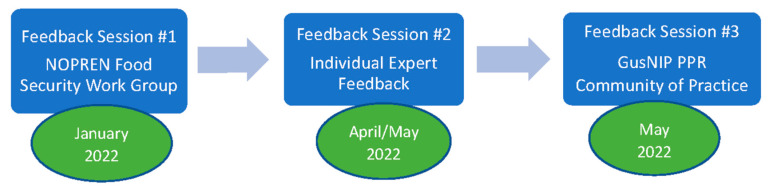
Graphic Representation of PPR TOC Development.

We applied a three-step process to adapt the NI TOC to develop the PPR TOC. This process is graphically represented in Figure 2. To create the PPR TOC, we sought iterative feedback and revisions on the NI TOC from food-as-medicine experts, individuals from the healthcare sector, and others involved specifically in GusNIP PPR program implementation, program evaluation, and leadership. These healthcare sector-based individuals had not been involved in the development of the NI TOC because the NI program does not include the healthcare sector.

**Step 1: Presentation of the NI TOC to NOPREN Food Security Work Group for feedback.** First, the NI TOC facilitators presented the NI TOC to the Centers for Disease Control and Prevention (CDC) Nutrition and Obesity Policy Research and Evaluation Network (NOPREN) food security workgroup in January 2022 [53]. We chose this workgroup because many members have expertise in food and nutrition security and financial incentives. After a short didactic presentation about generic TOC concepts and an overview of the NI TOC, facilitators used Jamboard, an online digital whiteboard that allows for group-based, real-time collaboration, and Zoom breakout rooms to moderate small group conversations around the questions “How should the PPR TOC be different than the NI TOC?” and, specifically, “What new pieces are needed for the PPR TOC, what pieces of the NI TOC should remain, and how should the NI TOC be modified to reflect the unique healthcare components of PPRs?” The presentation and workshop were facilitated by four expert consultants, three of whom are authors of this paper (HS, JK, KL). In total, 67 individuals attended this presentation, including nutrition policy researchers/evaluators, food security program implementers, advocates, CDC scientific staff, representatives from food banks, and representatives from health departments. We grouped the Jamboard feedback (n = 62 unique posted comments) into categories derived from the components of a TOC (e.g., pathways, outcomes, foundation). We used qualitative content analysis methods to determine categories and overarching key themes from these results [54]. Three of these themes were categorized by the headings required in TOC (e.g., pathways). We then used these findings to develop draft 1 of the PPR TOC, based on modifications to the NI TOC.

**Step 2: Expert feedback solicited (via email).** We next sought and received individual email-based feedback from four key informant experts in the fields of food and nutrition security; diversity, equity, and inclusion specific to food systems; and clinicians (MD, RDN). Between April and May 2022, one author (HS) sent a query email including the introduction and background of the TOC, draft 1 of the PPR TOC (e.g., NI TOC graphic [47] with some adaptations from NOPREN workshop), and four questions to elicit thoughts and perspectives from the experts. These questions included: “Is the content (e.g., overall goals, GusNIP outcomes, pathways of the TOC) accurate for the headings? Is anything missing? Does anything NOT belong? Can anything be condensed?” We summarized responses using qualitative content analysis methods [54] and made edits and modifications to the draft PPR TOC, yielding draft 2 of the PPR TOC.

**Step 3: Presentation of the newly adapted PPR TOC to GusNIP PPR Community of Practice.** The final method of seeking feedback was a 90 min online workshop in May 2022 at the GusNIP NTAE’s PPR Community of Practice. Forty individuals attended this workshop and included GusNIP PPR program implementers, external evaluators, and others involved with PPRs not funded by GusNIP. Two authors (HS, DN) provided a generic overview of TOCs and presented draft 2 of the PPR TOC. We then randomly assigned participants into breakout rooms with a facilitator (HS, MA, DN) in each room. Each breakout room focused on one element of the TOC and used Jamboard to elicit feedback using the same questions posed to the experts invited by email. These elements were TOC foundations, pathways, and equity. Because the PPR goals and outcomes were previously determined by USDA NIFA, we did not discuss them. Results from the Jamboard (n = 51 unique posted comments) were transferred to a word document and categorized by the different sections of the TOC. Researchers again employed qualitative content analysis principles to determine categories and overarching key themes from these results [51]. These findings were used to develop draft 3 of the PPR TOC.

Following the collection of feedback from the steps 1–3, the team compiled and synthesized all feedback received. Between June and October 2022, the team discussed the comments, addressed any outstanding questions, developed consensus regarding the PPR TOC content and prepared a revised version of the PPR TOC graphic image. We then engaged a scientific writer to review, edit content, and assist with refinement of the language and a graphic designer to adapt the NI TOC image for use in describing the PPR.

## 3. Results

A summary of those who participated in the three steps to inform development of the PPR TOC is depicted in Table 1.

Three overarching themes emerged from the feedback provided by the workgroups and key informants: (1) include health outcomes, (2) integrate a healthcare system pathway, and (3) emphasize equity. The NI TOC elicited a robust conversation about how healthcare representation was completely missing from the NI TOC. This is because GusNIP NI programs do not require partnerships with healthcare organizations, and therefore, they were justifiably absent from the NI TOC. Half (52%) of the Jamboard notes referred to the need to add the healthcare sector, health outcomes, and clinical components (e.g., role of healthcare providers) to the TOC. Other Jamboard responses included comments on pathways (11%), equity (16%), and goals and outcomes (21%). In response to these findings, we developed a first draft of the PPR TOC in which a new pathway was added (unique to PPR, as compared to the NI TOC)—“healthcare providers prescribe fruits and vegetables (FV) to eligible patients”—as well as new outcomes—“improved health outcomes and lower healthcare costs”.

The key informant experts reviewed draft 1 of the PPR TOC. Since this draft included new language on health outcomes and healthcare sector components, the experts focused on these elements of the TOC. They also addressed the presentation of the TOC graphic. Suggestions included redesigning the graphic visually, simplifying text, improving the accessibility of the image to comply with federal regulations that assure that people with disabilities can access information and communications (e.g., 508 compliance), and adding a glossary of terms. Clinician experts recommended fine-tuning the newly added healthcare outcome and cost language. Finally, two experts offered feedback on equity as it relates to shifting power and decision making to the communities that PPRs intend to serve.

After revising the PPR TOC draft to include the healthcare sector as a key component to PPR programming, subsequent feedback emphasized how equity should be depicted in the TOC. While each of the three breakout rooms in this workshop was assigned to focus on a different aspect of the TOC (e.g., pathways, foundations, equity), equity emerged as an important theme across all breakout rooms and accounting for 61% of all comments in the Jamboard. Informed by these findings, we edited the PPR TOC graphic to depict the importance of grounding all PPR TOC components, including foundations, pathways, outcomes, objectives, and goals, in equity using the image of the roots of a tree.

Several of the components of the final PPR TOC are similar to the NI TOC, such as overarching GusNIP program goals, which are predetermined by the requests for USDA NIFA GusNIP applications. The foundations of GusNIP are similar for NI and PPR programs, although the language describing them is condensed in the PPR TOC based on reviewer feedback. A key difference is the inclusion of healthcare goals, outcomes, and pathways in the PPR TOC. Equity is much more central and foundational in the PPR TOC graphic and serves as a basis for all other components. The NI TOC focus on “local” produce was removed from the PPR TOC because feedback suggested that eating FVs, regardless of source, is what contributes to improved health outcomes.

**Figure 3:**
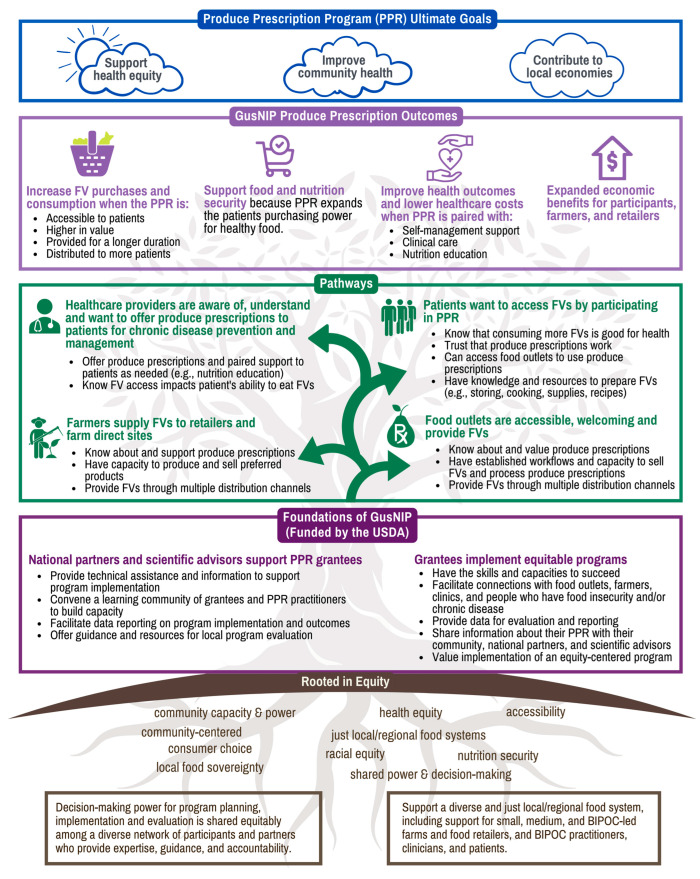
Produce Prescription Theory of Change Graphic.

The final PPR TOC diagram reflects these changes (Figure 3). The following narrative describes each component.

The GusNIP PPR is rooted in equity depicted by the roots of the tree. This means that decision-making power for all program planning, implementation, and evaluation is shared across a diverse network of participants and partners. Additionally, the GusNIP PPR centers around a diverse and just local system including small, medium, and BIPOC-owned farms and food retailers and BIPOC practitioners, clinicians, and patients/participants. The PPR commitment to equity is intentionally reflected throughout each of the TOC components.

Next in the PPR TOC are the pathways describing a strong GusNIP foundation depicted by the trunk of the tree. National partners, scientific advisors, and grantees partner to implement equitable PPR projects. The NTAE provides technical assistance to grantees, convenes a learning community of grantees and practitioners, and facilitates project reporting and evaluation. These supports provide grantees with the necessary skills, knowledge, and resources to succeed as they partner with communities to build successful, equity-centered projects.

With this foundation in place, grantees implement activities that generate four pathways leading to short-term outcomes (depicted by the branches of the tree):

1. **Healthcare providers are aware of, understand, and want to offer produce prescriptions to patients for chronic disease prevention and management.** Providers offer PPRs and paired support to patients as needed and know that FV access impacts a patient’s ability to consume FVs.
2. **Patients want FVs and want to participate in PPR projects.** Robust nutrition education offered by trusted clinic-based staff can facilitate this pathway.
3. **Food outlets are accessible, welcoming to diverse patrons, and provide FVs.** GusNIP PPR grants support food retailers to develop the infrastructure needed to process PPRs. They encourage participation by culturally diverse and locally owned and operated food retailer sites that are preferred shopping locations of participants because of locality and convenience.
4. **Farmers supply FVs to retailers and farm direct sites.** PPRs facilitate sales of FVs by a diverse network of farmers and food distributors to retailers.

These pathways lead to four short-term outcomes including increased FV purchases and intake; improved food security; improved health outcomes and lower healthcare costs; and expanded economic benefits for participants, farmers, and retailers. Finally, these outcomes, as supported by the foundations and pathways depicted in the TOC, ultimately contribute to the three main goals of the PPR: health equity, community health, and (similar to NI projects) supporting local economies.

## 4. Discussion

We developed a TOC for the GusNIP PPR through an iterative, participatory process. The TOC summarizes how and why the program works, identifies what the program hopes to achieve, and elucidates the causal pathways necessary to achieve its goals. The TOC will support current and future GusNIP grantees in grant writing, program implementation, and evaluation. It may also support USDA in understanding resources needed to support PPRs. We anticipate it will be useful for other PPR program implementers, evaluators, funders, and others interested in this evolving area as well.

The GusNIP PPR funding has catalyzed the development of local programs across the US. It has supported 44 awards to 30 unique grantees across 20 states and the District of Columbia in fiscal years 2019, 2020, and 2021, with grants ranging from USD 129,018 to USD 647,027 [41]. Additionally, in 2022, The American Rescue Plan Act (ARPA) funded 72 PPR awards ranging from USD 80,839 to USD 500,000 to 72 grantee organizations across 33 states and DC. Data from a small sample of participants provide preliminary evidence to suggest that they have increased intake of FVs, supported food security, and improved health status [41,55].

The TOC described in this manuscript builds on the rigorous, participatory, equity-focused work conducted to develop the TOC for the GusNIP NI program. NIs provide financial incentives for purchases of FVs, specifically among people participating in SNAP, and are intended to support improved dietary intake and food security and generate local economic impact. Due to the similarities in NI and PPR programs, we were able to leverage the NI TOC as a starting point for the development of the PPR TOC. In particular, the foundations of the GusNIP program, many of the pathways, and some of the outcomes and ultimate goals were highly similar. Thus, the initial version of the PPR TOC was virtually identical to the NI TOC and allowed participants to specifically call attention to ways in which the programs operated differently.

We found that for some TOC components, our assumptions about similarities between the two programs held, and few adaptations were needed to repurpose the NI TOC elements for use in the PPR TOC. However, major changes were needed for components that differed substantially between NI and PPR programs: integration of PPRs within healthcare settings and an explicit focus on improvement in health outcomes. In addition, new ideas and perspectives on how equity should appear in the TOC emerged, which were reflected in innovative wording and framing and, most importantly, in a new graphic design which positions equity as the roots of, and thus fundamental to, the rest of the process described in the PPR TOC.

Unlike NI programs, PPR programs are closely integrated with the healthcare system. The lead agency implementing a PPR program is often a healthcare system (e.g., federally qualified healthcare center or hospital), or a healthcare system may be a partner to a non-clinical grantee (e.g., farmers market association). The clinical organization enrolls participants and/or distributes the fruit and/or vegetable resource (e.g., a food box, a voucher, a debit card, etc.). Thus, the TOC had to include an entirely new partner and sector, reflected in a new pathway focused on healthcare providers, suggesting THAT healthcare providers prescribe fruits and vegetables to eligible patients. Other pathways describe sectors and stakeholders that are similar for NIs: farmers, retailers, farm direct sites, and program participants. Much of the discussion about the inclusion of the healthcare pathway centered on the complexity of the healthcare system and the challenges it has coordinating activities with the other sectors necessary for developing a PPR program. The final TOC healthcare system pathway elevated the need for system capacity to identify eligible patients, co-intervention components such as nutrition education, and clinician knowledge of both PPR programming and the importance of increased FV intake. A number of studies on PPR implementation affirm the importance of these barriers and facilitators to successful implementation in the healthcare setting [56,57,58].

Other consequences of adding a healthcare focus to the TOC were more straightforward, reflecting ways in which PPRs differ from NI programs. For example, PPRs use the word “patients”, rather than “participants”, to describe program enrollees. The PPR outcome articulated in the TOC as “improved health outcomes and costs” is justifiably not included in the NI TOC. This outcome is consistent with the objectives articulated in the legislation establishing the GusNIP program: “the improvement of dietary health and the reduction in healthcare use and associated costs” [59].

Equity was a topic of much discussion in development of the TOCs for both NI and PPR programs. Equity is depicted as more foundational in the PPR TOC, as many of the PPR TOC key informant interviewees noted the importance of explicitly grounding the TOC in equity, rather than it being a siloed construct that may not be fully woven into all aspects of the TOC. It may be that since NI programs are inherently reaching communities with limited income (e.g., people who participate in SNAP), equity was even more prominent in the PPR TOC discussions. Further, unlike NI programs where users must participate in SNAP to receive NI benefits, SNAP use is not a prerequisite for participating in PPRs, and thus, equitably establishing that people who participate in PPRs need the benefit is critical. Further, PPRs are one example of “Food as Medicine” programs, which have central goals to promote equity and decrease health disparities. Food as Medicine programs have gained attention and traction in the last few years (e.g., White House Conference on Hunger, Health, and Nutrition occurred in September 2022, which had Food as Medicine and health equity prominently featured), so the concept of equity may have been at the forefront as participants were thinking about relevant PPR TOC components.

This TOC is designed to aid healthcare organizations and their partners, program implementers, policymakers, researchers, and funders as they design, implement, evaluate, and fund PPR programs. Essentially, it describes current understanding of the mechanisms, assumptions, rationale, and underpinnings that lead to successful and equitable outcomes within the GusNIP PPR. The PPR TOC provides a framework for implementation that can facilitate collaboration and decision making when developing or sustaining a project. For example, staff that want to champion a PPR project may use the TOC to inform a request to clinic administration to apply for GusNIP or other funding. The TOC can be used during implementation of a PPR program. It can help onboard partners, develop workflows, and establish data processes. Researchers can crosswalk the PPR TOC with current evidence to identify well-studied areas (e.g., increased FV intake) as well as gaps (e.g., healthcare cost and utilization). Funders and policymakers can use the PPR TOC to guide language for funding requests for applications and policy. Pillar 2 of the Biden–Harris Administration National Strategy on Hunger, Nutrition, and Health calls for actions to expand produce prescriptions, and this TOC can be used to establish projects that do not reinvent the wheel but rather build upon existing work [60].

### Strengths and Limitations

The approach used to develop this PPR TOC has both strengths and limitations. Leveraging the robust work this team and others had done to develop the NI TOC allowed us to develop the PPR TOC efficiently and cost-effectively. We used input collected from preparation of the NI TOC to inform development of the PPR TOC. This allowed us to minimize the data collection burden on the invested parties who participated in the PPR TOC process. The reduction of burden is important in a relatively small field of invested parties, many of whom are working under considerable resource constraints and for whom time is an increasingly valuable commodity. However, this process may have predisposed our PPR TOC to being more like the NI TOC than if we had started from scratch. In addition, we relied on the NI TOC process to bring in many perspectives (including those of program participants, food retail representatives, and farming experts) that we were unable include in the development of the PPR TOC given resource constraints.

Additional strengths of our process included the integration of multiple sources of information in a document and evidence review, several engagement modalities (workshops, email) to engage informants, and the subject matter expertise of the facilitators. Although informants participated virtually and were often in mixed settings where power imbalances sometimes emerge, we used collaboration tools (such as Jamboard) so that members had a choice in whether to contribute orally or using written text. Such flexibility encourages more equal opportunity for participation.

Like the NI TOC, this PPR TOC is designed to be readily understood by people without extensive expertise in nutrition programs and GusNIP. Thus, it omits many details that might be included in a more comprehensive TOC targeted to an academic audience. In addition, because of the complexity of the broader food system, we had to simplify and streamline many components of the pathways.

Because we focused specifically on PPRs funded by GusNIP, this TOC may not be generalizable to PPRs funded by other sources. In addition, PPRs—even those funded by GusNIP—are often small, locally tailored, and rooted in the culture and workflows of a specific healthcare organization. Due to this heterogeneity, a PPR may find that this TOC needs minor adaptation for its specific context.

Finally, evidence on the impact of PPRs and the mechanisms leading to impacts is still relatively immature. Data supporting some of the TOC pathways and outcomes are limited. As more data emerge, this TOC will need to evolve to incorporate new findings. There were many suggestions for how to advance equity in PPRs but very little evidence available to suggest which strategies are most likely to result in more equitable outcomes. This is an important area of future research. The growing number of PPRs will generate practice-based evidence that will be useful in refining the TOC. Iterative updating of the TOC as new knowledge is generated about how to achieve successful and equitable outcomes within the GusNIP PPR program is essential. TOCs are living documents that evolve along with the field.

## 5. Conclusions

We adapted the TOC for GusNIP NI projects to be relevant for GusNIP PPRs and other-funded PPRs. Modifications focused on three areas where PPRs differ from NI programs: (1) inclusion of healthcare as an additional partner, (2) the importance of health and healthcare utilization as outcomes, and (3) equity considerations. The PPR TOC will be useful for produce prescription developers, practitioners, funders, and evaluators for describing the pathways, assumptions, and foundations of successful PPRs.

## Figures and Tables

**Table 1:** Produce Prescription Programs (PPRs) Theory of Change (TOC) Contributors, Roles, and Number of Participants for Each Engagement Method.

Type of Contributor (n)	Role	Workshop Attendees ^a^ (n = 113)	Email Feedback ^b^ (n = 4)
Gus Schumacher Nutrition Incentive Program (GusNIP) Training, Technical Assistance, Evaluation, and Information Center (NTAE) (6)	Collaborated with facilitators to develop the TOC. Brought partners and practitioners to the TOC process. Contributed deep knowledge about the history of GusNIP, how the program works, and the operation of the NTAE.	6	0
Partners (70)	NTAE partners and additional expert advisors from agriculture, food retail, academic, food security, healthcare, and nutrition sectors. Brought expertise in PPR program impacts and best practices.	67	3
Practitioners (41)	GusNIP-funded PPRs and other practitioners with broad experience in produce prescription. Brought extensive knowledge about how PPRs are implemented.	40	1

^a^ During development of the PPR TOC, two workshops (67 partners in workshop #1 and 40 practitioners in workshop #2) occurred to develop a common understanding of the TOC, introduce and refine the PPR TOC, and ensure an equity lens contributed to TOC development. ^b^ Targeted emails were sent to identified produce prescription experts (n = 4) seeking feedback on the PPR TOC.

## Data Availability

Not applicable.

## References

[1] Boeing H., Bechthold A., Bub A., Ellinger S., Haller D., Kroke A., Leschik-Bonnet E., Müller M.J., Oberritter H., Schulze M. (2012). Critical Review: Vegetables and Fruit in the Prevention of Chronic Diseases. Eur. J. Nutr..

[2] Aune D., Giovannucci E., Boffetta P., Fadnes L.T., Keum N.N., Norat T., Greenwood D.C., Riboli E., Vatten L.J., Tonstad S. (2017). Fruit and Vegetable Intake and the Risk of Cardiovascular Disease, Total Cancer and All-Cause Mortality-A Systematic Review and Dose-Response Meta-Analysis of Prospective Studies. Int. J. Epidemiol..

[3] Hu D., Huang J., Wang Y., Zhang D., Qu Y. (2014). Fruits and Vegetables Consumption and Risk of Stroke: A Meta-Analysis of Prospective Cohort Studies. Stroke.

[4] Wang L., Manson J., Gaziano M., Buring J., Sesso H. (2012). Fruit and Vegetable Intake and the Risk of Hypertension in Middle-Aged and Older Women. Am. J. Hypertens..

[5] Li M., Fan Y., Zhang X., Hou W., Tang Z. (2014). Fruit and Vegetable Intake and Risk of Type 2 Diabetes Mellitus: Meta-Analysis of Prospective Cohort Studies. BMJ Open.

[6] Stanaway J.D., Afshin A., Ashbaugh C., Bisignano C., Brauer M., Ferrara G., Garcia V., Haile D., Hay S.I., He J. (2022). Health Effects Associated with Vegetable Consumption: A Burden of Proof Study. Nat. Med..

[7] Xu J., Murphy S., Kochanek K., Arias E. (2022). Mortality in the United States, 2021.

[8] Lee Y., Mozaffarian D., Sy S., Huang Y., Liu J., Wilde P.E., Abrahams-Gessel S., Veiga Jardim T.D.S., Gaziano T.A., Micha R. (2019). Cost-Effectiveness of Financial Incentives for Improving Diet and Health through Medicare and Medicaid: A Micro Simulation Study. PLoS Med..

[9] U.S. Department of Agriculture, U.S. Department of Health and Human Services (2020). Dietary Guidelines for Americans, 2020–2025.

[10] Lee S.H., Latetia, Moore V., Park S., Harris D.M., Blanck H.M. (2022). Morbidity and Mortality Weekly Report Adults Meeting Fruit and Vegetable Intake Recommendations-United States, 2019.

[11] Centers for Disease Control and Prevention (2018). State Indicator on Fruits and Vegetables, 2018.

[12] Herforth A., Ahmed S. (2015). The Food Environment, Its Effects on Dietary Consumption, and Potential for Measurement within Agriculture-Nutrition Interventions. Food Secur..

[13] Wang Y., Beydoun M.A. (2007). The Obesity Epidemic in the United States—Gender, Age, Socioeconomic, Racial/Ethnic, and Geographic Characteristics: A Systematic Review and Meta-Regression Analysis. Epidemiol. Rev..

[14] Sallis J.F., Glanz K. (2009). Physical Activity and Food Environments: Solutions to the Obesity Epidemic. Milbank Q..

[15] Fleischhacker S.E., Evenson K.R., Rodriguez D.A., Ammerman A.S. (2011). A Systematic Review of Fast Food Access Studies. Obes. Rev..

[16] Byker Shanks C., Ahmed S., Smith T., Houghtaling B., Jenkins M., Margetts M., Schultz D., Stephens L. (2015). Availability, Price, and Quality of Fruits and Vegetables in 12 Rural Montana Counties, 2014. Prev. Chronic Dis. Public Health Res. Pract. Policy.

[17] Larson N.I., Story M.T., Nelson M.C. (2009). Neighborhood Environments: Disparities in Access to Healthy Foods in the U.S. Am. J. Prev. Med..

[18] Whiteman E.D., Chrisinger B.W., Hillier A. (2018). Diet Quality Over the Monthly Supplemental Nutrition Assistance Program Cycle. Am. J. Prev. Med..

[19] Dong D., Lin B.H. (2011). Consumption by Low-Income Americans: Would a Price Reduction Make a Difference?. Eating Right: The Consumption of Fruits and Vegetables.

[20] Young S.K., Stewart H.U.S. (2022). Fruit and Vegetable Affordability on the Thrifty Food Plan Depends on Purchasing Power and Safety Net Supports. Int. J. Environ. Res. Public Health.

[21] Glanz K., Basil M., Maibach E., Goldberg J., Snyder D. (1998). Why Americans Eat What They Do: Taste, Nutrition, Cost, Convenience, and Weight Control Concerns as Influences on Food Consumption. J. Am. Diet. Assoc..

[22] Kern D.M., Auchincloss A.H., Stehr M.F., Roux A.V.D., Moore L.V., Kanter G.P., Robinson L.F. (2017). Neighborhood Prices of Healthier and Unhealthier Foods and Associations with Diet Quality: Evidence from the Multi-Ethnic Study of Atherosclerosis. Int. J. Environ. Res. Public Health.

[23] Rao M., Afshin A., Singh G., Mozaffarian D. (2013). Do Healthier Foods and Diet Patterns Cost More than Less Healthy Options? A Systematic Review and Meta-Analysis. BMJ Open.

[24] Roberto C.A., Swinburn B., Hawkes C., Huang T.T.-K., Costa S.A., Ashe M., Zwicker L., Cawley J.H., Brownell K.D. (2015). Patchy Progress on Obesity Prevention: Emerging Examples, Entrenched Barriers, and New Thinking. Lancet.

[25] De Marchis E.H., Torres J.M., Benesch T., Fichtenberg C., Allen I.E., Whitaker E.M., Gottlieb L.M. (2019). Interventions Addressing Food Insecurity in Health Care Settings: A Systematic Review. Ann. Fam. Med..

[26] Engel K., Ruder E.H. (2020). Fruit and Vegetable Incentive Programs for Supplemental Nutrition Assistance Program (SNAP) Participants: A Scoping Review of Program Structure. Nutrients.

[27] Verghese A., Raber M., Sharma S. (2019). Interventions Targeting Diet Quality of Supplemental Nutrition Assistance Program (SNAP) Participants: A Scoping Review. Prev. Med..

[28] Gneezy U., Meier S., Rey-Biel P. (2011). When and Why Incentives (Don’t) Work to Modify Behavior. J. Econ. Perspect..

[29] Mozaffarian D., Rogoff K.S., Ludwig D.S. (2014). The Real Cost of Food: Can Taxes and Subsidies Improve Public Health?. JAMA.

[30] John S., Lyerly R., Wilde P., Cohen E.D., Lawson E., Nunn A. (2021). The Case for a National SNAP Fruit and Vegetable Incentive Program. Am. J. Public Health.

[31] Yaroch A.L., Byker Shanks C., Nugent N.B., Fricke H.E., Parks C.A. (2022). Potential of Financial Incentives to Promote Fruit and Vegetable Intake and Support Food Insecurity. U. N. Nutr. J..

[32] Andreyeva T., Long M.W., Brownell K.D. (2010). The Impact of Food Prices on Consumption: A Systematic Review of Research on the Price Elasticity of Demand for Food. Am. J. Public Health.

[33] Blakely T., Ni Mhurchu C., Jiang Y., Matoe L., Funaki-Tahifote M., Eyles H.C., Foster R.H., McKenzie S., Rodgers A. (2011). Do Effects of Price Discounts and Nutrition Education on Food Purchases Vary by Ethnicity, Income and Education? Results from a Randomised, Controlled Trial. J. Epidemiol. Community Health.

[34] Epstein L.H., Jankowiak N., Nederkoorn C., Raynor H.A., French S.A., Finkelstein E. (2012). Experimental Research on the Relation between Food Price Changes and Food-Purchasing Patterns: A Targeted Review. Am. J. Clin. Nutr..

[35] French S.A., Jeffery R.W., Story M., Breitlow K.K., Baxter J.S., Hannan P., Snyder M.P. (2001). Pricing and Promotion Effects on Low-Fat Vending Snack Purchases: The CHIPS Study. Am. J. Public Health.

[36] Gittelsohn J., Rowan M., Gadhoke P. (2012). Interventions in Small Food Stores to Change the Food Environment, Improve Diet, and Reduce Risk of Chronic Disease. Prev. Chronic Dis..

[37] Song H.-J., Gittelsohn J., Kim M., Suratkar S., Sharma S., Anliker J. (2009). A Corner Store Intervention in a Low-Income Urban Community Is Associated with Increased Availability and Sales of Some Healthy Foods. Public Health Nutr..

[38] Bartlett S., Klerman J., Olsho L. (2014). Healthy Incentives Pilot (HIP): Final Report.

[39] Vericker T., Dixit-Joshi S., Giesen L. (2021). Evaluation of the Implementation of Food Insecurity Nutrition Incentives (FINI): Final Report.

[40] USDA, National Institute of Food and Agriculture (2020). Gus Schumacher Nutrition Incentive Program.

[41] GusNIP, National Technical Assistance and Evaluation Center (2023). Gus Schumacher Nutrition Incentive Program (GusNIP): Impact Findings Y3: September 1, 2021 to August 31, 2022.

[42] Stotz S.A., Thompson J.J., Bhargava V., Scarrow A., Capitano K., Lee J.S. (2019). A Supplemental Produce and ELearning Nutrition Education Program for Georgians Who Use Safety-Net Clinics for Their Health Care. J. Nutr. Educ. Behav..

[43] Jones L.J., VanWassenhove-Paetzold J., Thomas K., Bancroft C., Quinn Ziatyk E., Kim L.S.H., Shirley A., Warren A.C., Hamilton L., George C.V. (2020). Impact of a Fruit and Vegetable Prescription Program on Health Outcomes and Behaviors in Young Navajo Children. Curr. Dev. Nutr..

[44] Aiyer J.N., Raber M., Bello R.S., Brewster A., Caballero E., Chennisi C., Durand C., Galindez M., Oestman K., Saifuddin M. (2019). A Pilot Food Prescription Program Promotes Produce Intake and Decreases Food Insecurity. Transl. Behav. Med..

[45] Budd Nugent N., Byker Shanks C., Seligman H., Fricke H., Parks C., Stotz S., Yaroch A. (2021). Accelerating Evaluation of Financial Incentives for Fruits and Vegetables: A Case for Shared Measures. Int. J. Environ. Res. Public Health.

[46] Nutrition Incentive Hub. https://www.centerfornutrition.org/gusnip.

[47] Leng K.H., Yaroch A.L., Nugent N.B., Stotz S.A., Krieger J. (2022). How Does the Gus Schumacher Nutrition Incentive Program Work? A Theory of Change. Nutrients.

[48] Center for Theory of Change (2023). What Is Theory of Change?.

[49] Mayne J. (2017). Theory of Change Analysis: Building Robust Theories of Change. Can. J. Progr. Eval..

[50] Harries E., Hodgson L., Noble J. (2014). Creating Your Theory of Change: NPC’s Practical Guide.

[51] HM Treasury Magenta Book *Central Government Guidance on Evaluation*; 2020; ISBN 9781913635183. https://assets.publishing.service.gov.uk/government/uploads/system/uploads/attachment_data/file/879438/HMT_Magenta_Book.pdf.

[52] Breuer E., Lee L., De Silva M., Lund C. (2016). Using Theory of Change to Design and Evaluate Public Health Interventions: A Systematic Review. Implement. Sci..

[53] University of California San Francisco—Center for Vulnerable Populations (2023). Nutrition & Obesity Policy Research & Evaluation Network (NOPREN).

[54] Hsieh H.-F., Shannon S.E. (2005). Three Approaches to Qualitative Content Analysis. Qual. Health Res..

[55] Gretchen Swanson Center for Nutrition Gus Schumacher (2021). Nutrition Incentive Program Training, Technical Assistance, Evaluation, and Information Center (GusNIP NTAE): Impact Findings.

[56] Auvinen A., Simock M., Moran A. (2022). Integrating Produce Prescriptions into the Healthcare System: Perspectives from Key Stakeholders. Int. J. Environ. Res. Public Health.

[57] Newman T., Lee J.S. (2021). Strategies and Challenges: Qualitative Lessons Learned from Georgia Produce Prescription Programs. Health Promot. Pract..

[58] Stotz S.A., Budd Nugent N., Ridberg R., Byker Shanks C., Her K., Yaroch A.L., Seligman H. (2022). Produce Prescription Projects: Challenges, Solutions, and Emerging Best Practices—Perspectives from Health Care Providers. Prev. Med. Rep..

[59] Conaway M.K. (2018). H.R.2—Agriculture Improvement Act of 2018.

[60] (2022). Biden Harris Administration Biden-Harris Administration National Strategy on Hunger, Nutrition, and Health.

